# Case report: late perianal mucinous adenocarcinoma after Crohn's disease proctectomy: an oncological rarity

**DOI:** 10.1186/1477-7819-3-42

**Published:** 2005-06-29

**Authors:** Michael Keese, Walter Back, Dietmar Dinter, Rainer Gladisch, Andreas Joos, Pablo Palma

**Affiliations:** 1 Department of Surgery, University Hospital of Mannheim, 68135 Mannheim, Germany; 2 Department of Pathology, University Hospital of Mannheim, 68135 Mannheim, Germany; 3 Department of Radiology, University Hospital of Mannheim, 68135 Mannheim, Germany; 4 Department of Medicine, University Hospital of Mannheim, 68135 Mannheim, Germany

## Abstract

**Background:**

As in ulcerative colitis, there is an increased incidence of colorectal carcinoma in Crohn's disease. While carcinoma formation originating from ano-rectal fistulas is generally considered as a rare event there are different publications reporting on mucinous adenocarcinoma formation in association with a neovagina and rectovaginal fistulas. To the best of our knowledge this is the first description of a perianal mucinous adenocarcinoma arising in a patient after Crohn's disease proctocolectomy.

**Case presentation:**

We report the case of a 50-year old female with a mucinous adenocarcinoma forming in the perineum eleven years after proctocolectomy for Crohn's disease. The patient was readmitted with perineal pain, leucocytosis and a perineal mass highly suspicious of abscess formation in the MRI-Scan. Histological examination revealed a mucinous adenocarcinoma. Exenteration including vagina, uterus and ovaries together with the coccygeal-bone was performed.

**Conclusion:**

Mucinous adenocarcinoma formation is a rare complication of Crohn's disease and so far unreported after proctocolectomy.

## Background

As in ulcerative colitis, there is an increased incidence of colorectal carcinoma in Crohn's disease [1]. An increased risk of cancer in patients with Crohn's disease has been shown to be related to an early onset and a prolonged duration of the inflammatory bowel disease [2]. Furthermore, several cases have been reported in which carcinoma formation originated from ano-rectal fistulas which are commonly associated with Crohn's disease [3]. While carcinoma formation originating from anorectal fistulas is generally considered as a rare event, these tumors are most commonly either mucinous carcinomas or squamous cell carcinomas [4]. The causative relationship between anorectal fistulas and cancer is not known. Cancer development in these cases remains a diagnostic challenge especially if carcinomas arise in the midst of abscess formation [5]. We report late formation of a mucinous adenocarcinoma in a patient with Crohn's disease who presented severe perineal fistulous lesions after proctocolectomy.

## Case presentation

In October 2004 a 50-year old woman was admitted presenting with gluteal and perineal fistula formation after proctocolectomy eleven years earlier. The clinical examination showed a single fistula opening on the perineum with purulent secretion. A second opening was found on the posterior wall of the vagina.

**Figure 1:**
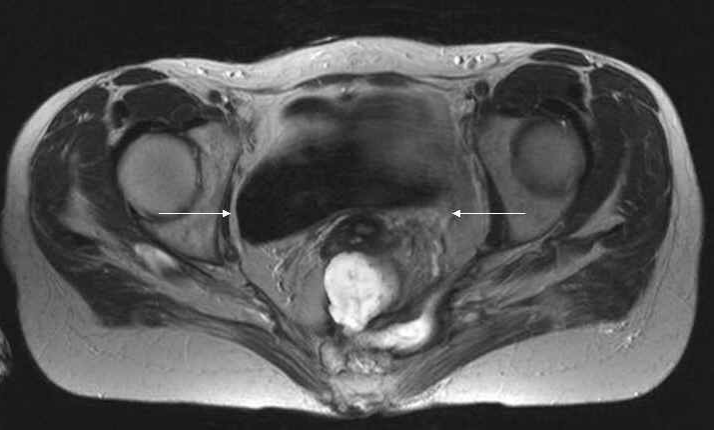
A T2-weighted axial MR image of the pelvis showing an irregular presacral fluid collection (arrows) extending from the cervix to the coccyx and to the sciatic foramen on the left side.

**Figure 2:**
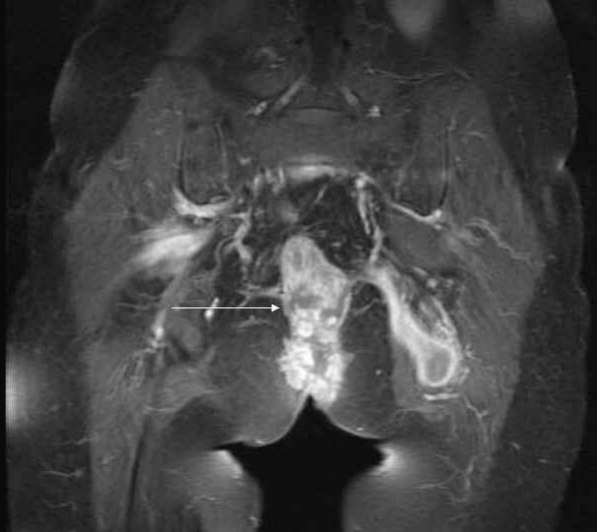
T1- weighted fat saturated MR image after i.v. application of contrast medium coronal. The arrow is pointing to a fistulating formation spreading in the gluteal muscles on the left side with typical rim enhancement and central liquid components; the soft tissue mass seen in midline has a centrally necrotizing appearance.

**Figure 3:**
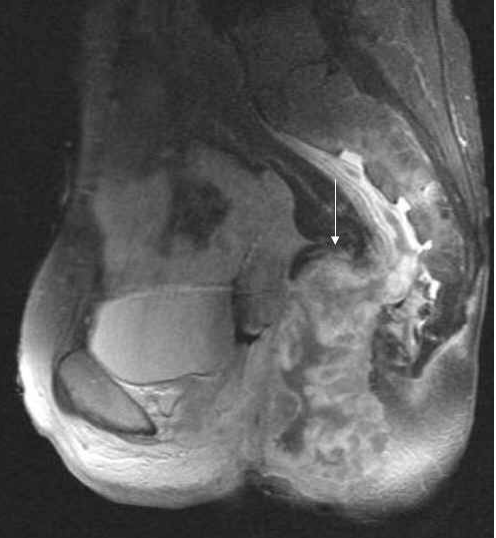
T1- weighted fat saturated MR image after i.v. application of contrast medium sagittal view. Presacrally, the mass characterized by an inhomogeneous contrast medium uptake in the thickened, nodular wall spreads in the subperiosteal space, as indicated.

The MRI-scanning revealed a large formation in the lower pelvis reaching to the sacrum. This mass showed a thickened, contrast-enhancing wall and necrotic, liquid inner parts suggestive of abscess-formation. No involvement of the small bowel or bladder was detected (Figures 1, 2, 3).

The patient's history revealed Crohn's disease first diagnosed in 1981. Ten years later (1991), the patient was readmitted because of severe perineal fistula disease. The clinical and radiological examination showed multiple transphincteric fistulas and a single rectovaginal fistula as well as one enterocutaneous fistula originating from the terminal ileum. After medical management (parenteral nutrition and corticoids) she was referred to the department of surgery where an ileocoecal resection with a diversion ileostomy and drainage of the perianal fistulas were performed. Postoperatively the patient developed all signs of an Addison crisis, which could be satisfactorily treated.

The patient was readmitted in 1993 with a new inflammatory episode including perianal fistulating disease. Because of the disease recurrence and due to further impairment of the sphincter mechanism a proctocolectomy with exstirpation of the sphincter ani muscles and a terminal ileostomy was recommended and performed. The histopathological examination showed signs of Crohn's disease with some epitheloid cell granulomas and giant cells as well as microgranulomas in both the large bowel and the rectum. No signs of malignancy and no dysplastic changes were found. The patient developed a paralytic ileus that could be treated conservatively and a perianal wound infection which required secondary wound closure.

Three years later, in 1996, the patient was readmitted because of a persistent perineal secretion. No fistulas and no signs of Crohn's disease could be found in the small bowel. The patient was treated with local wound therapy without any further surgical procedure until readmission in 2004.

### Surgical therapy

After informed consent a perineal drainage with entire resection of the clinically inflamed tissue was performed. Intraoperatively the tissue showed a colloidal consistency and fistulation into the coccygeal bone and the vagina. Histological analysis of the tissue detected a mucinous adenocarcinoma occupying the resected tissue and the resection margins. The tumour consisted of moderately atypical glandular cell elements lying in pools of PAS-positive mucin. A distinct fibroblastic stromal reaction could be found in the surrounding mesenchyma.

Upon diagnosis, a second intervention was performed including an en-bloc exenteration of the uterus, vagina and ovaries via laparotomy and resection of the sacrum through a posterior approach. The pelvis was closed using a Vypro^® ^mesh (polypropylene-polygalactine composite) and the perineum was left open for secondary wound healing.

Postoperatively, bladder function was impaired, otherwise the patient recovered well and could be dismissed into out patient oncological care.

### Histopathology

**Figure 4:**
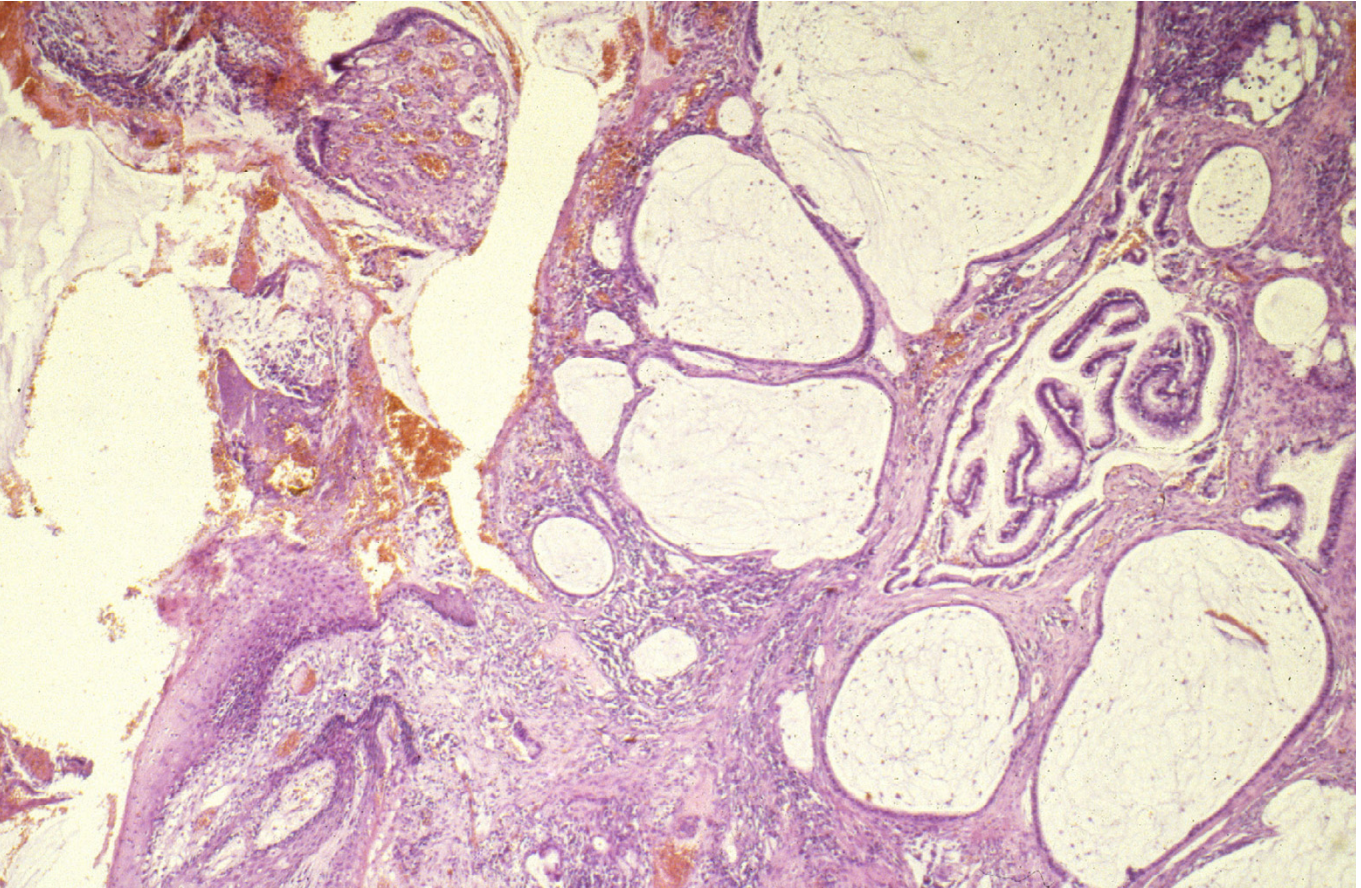
Histopathological overview of the fistula opening on the perineum consisting of mucus containing cystic spaces, partially lined by cylindrical epithelium of the mucinous adenocarcinoma. H&E, × 25.

**Figure 5:**
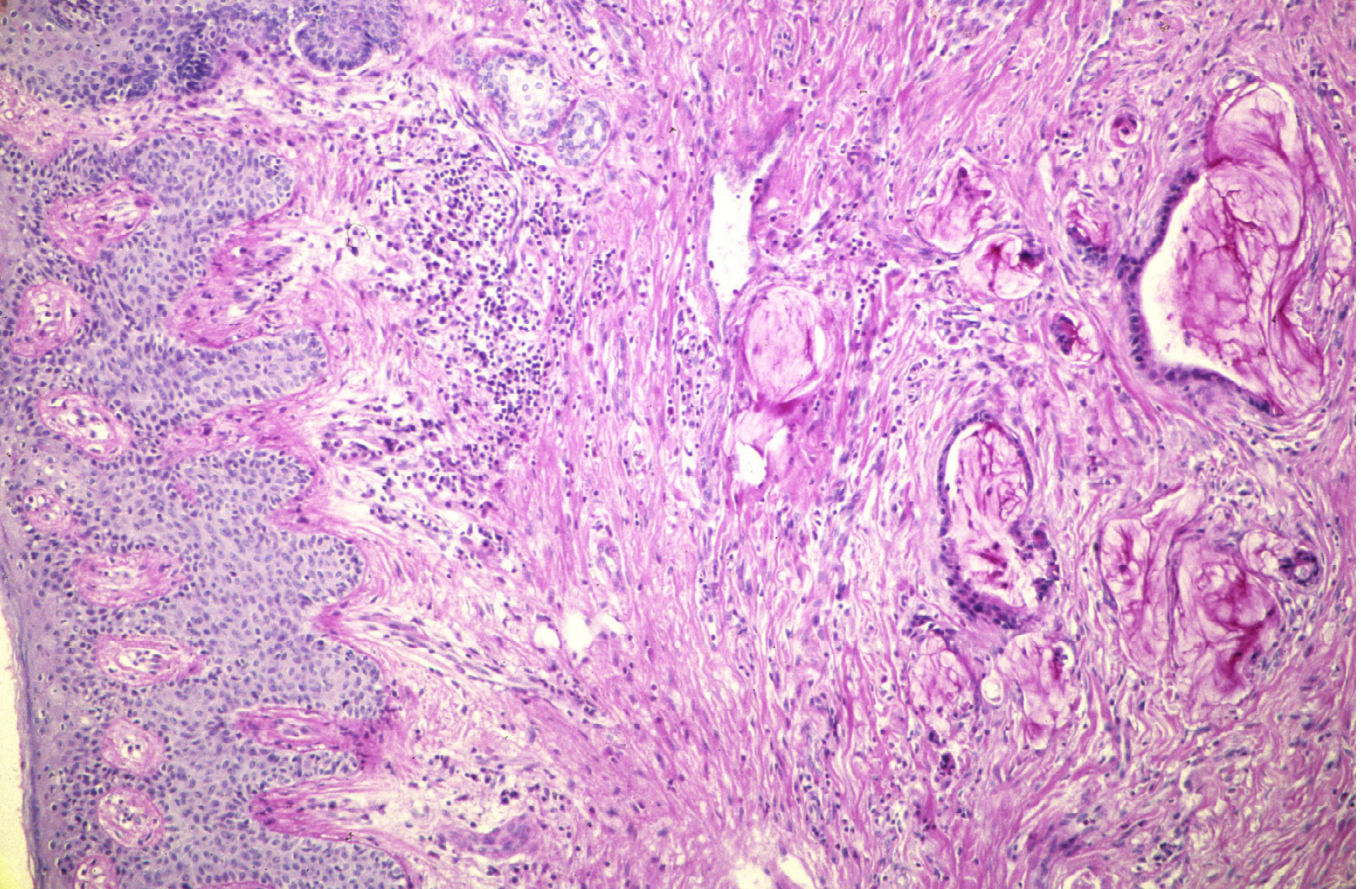
Dermal infiltration of the mucinous adenocarcinoma undermining the perineal skin. PAS-stain, × 50.

Examination of the specimen showed typical cylindrical epithelium of colorectal type lining some residual lumina of fistulas and cysts (Figure 4). The resection margins did not show any residual fistulas. No rests of original bowel mucosa or remaining bowel wall structures could be found in the "en-bloc" specimen. But there were some minor residual carcinomatous infiltrates in the soft tissues between coccygeal bone and dorsal wall of the vagina (Figure 5). Bone tissue and the vaginal wall structures themselves proofed to be free of tumour histologically. No florid, ulcerating or granulomatous inflammation was found in the remaining mucosal tissues.

## Discussion

Despite the original Crohn's disease description in 1932, it was not until 1938 that Penner and Crohn described the presence of a perianal fistula in a patient with Crohn's disease [6]. Isolated perianal involvement has been reported in fewer than 5% of cases [7]. Patients with colonic involvement are most likely to develop perianal Crohn's disease, seen in 46% to 68% of this patient group, and 5% to 27% of patients with small bowel disease develop perianal lesions [8].

Surgical options for perianal Crohn's disease range from abscess drainage as ''first-aid management'' to major interventions [9], such as proctocolectomy and permanent stoma formation as in the presented case. A proctocolectomy was performed in this patient because of severe and recurrent perineal disease that did not respond to local surgery combined with medical treatment. However, among colorectal surgeons poor wound healing and perineal sinus formation are well recognized complications after proctectomy.

As for ulcerative colitis, patients with longstanding Crohn's disease are at increased risk (3.7 per cent) of developing adenocarcinoma. This has been attributed to an early onset and prolonged duration of disease. The incidence of carcinoma is 0.7 per cent in patients with perineal Crohn's disease; both adenocarcinomas and squamous cell carcinomas occur [4,10].

For mucinous adenocarcinoma the overall incidence among all colorectal carcinomas is ranging from 7.8 per cent to 18 per cent. As a rare tumor entity among sporadic colon carcinomas, these tumors are most frequently found in the right-sided colon followed by the rectum and tend to be associated with an inflammatory process, such as colitis, ulcerative colitis and Crohn's disease [12,13]. As assessed by our histopathological studies no residual rectal mucosa was found in the perineum. In the en-bloc specimen signs of persistant perineal inflammatory Crohn's disease were found. There are several publications reporting this kind of adenocarcinoma in association with a neovagina and rectovaginal fistulas [14-16]. Pathogenetically, perianal mucinous adenocarcinoma are thought to arise from the anal ducts if triggered by chronic inflammation [13]. One single case of mucinous carcinoma development in a patient with persistant perineal crohn's disease has been described after ileorectal anastomosis. Here the tumor had formed within the fistula [17]. In our case histomorphologically, it cannot be decided if the tumour developed from residual perianal gland mucosa or has evolved in residual perianal fistulas.

To the best of our knowledge, this is the first case report of a late perianal mucinous adenocarcinoma arising in a patient after proctocolectomy for Crohn's disease. The present case should make surgeons and gastroenterologist aware of the risk of poor healing and the associated morbidity after rectal excision performed for perianal Crohn's disease in order to consider alternative therapeutical options. As a possible alternative, the literature shows that a low Hartmann's procedure may result in a 60 per cent healing rate in patients with perineal disease [18]. However, up to 50 per cent of these patients (perineal Crohn's disease treated by Hartmann's procedure) required a completion proctectomy, because they showed residual disease in the rectal stump [19]. Diversion ileostomy before proctocolectomy, which may convert active disease to a quiescent state, could be another effective alternative in order to avoid sinus development. There are also favorable reports about the use of gracilis transposition flap to treat perineal wounds after proctectomy [20].

The presented case with cancer formation shows, that even a radical surgical approach to treat perineal fistulous disease does not withhold late complications of chronic perineal inflammatory Crohn's disease. Moreover, this report should alert surgeons to avoid a perineal sinus or poor wound healing after proctectomy. Alternative surgical procedures combined with optimized medical therapy should be preferred over proctectomy in Crohn's disease.
